# Bacterial divergence among the interconnected habitats of a High Arctic Lake

**DOI:** 10.1093/femsec/fiaf115

**Published:** 2025-11-18

**Authors:** Pénélope Blackburn-Desbiens, Maxime Larose, Raoul-Marie Couture, Warwick F Vincent, Alexander I Culley, Catherine Girard

**Affiliations:** Département des sciences fondamentales, Université du Québec à Chicoutimi, Chicoutimi, Québec G7H 2B1, Canada; Centre d’études nordiques (CEN), Université Laval, Québec, Québec G1V 0A6, Canada; Groupe de recherche interuniversitaire en limnologie (GRIL), Montreal, Québec H3C 3J7, Canada; Département des sciences fondamentales, Université du Québec à Chicoutimi, Chicoutimi, Québec G7H 2B1, Canada; Centre d’études nordiques (CEN), Université Laval, Québec, Québec G1V 0A6, Canada; Groupe de recherche interuniversitaire en limnologie (GRIL), Montreal, Québec H3C 3J7, Canada; Centre d’études nordiques (CEN), Université Laval, Québec, Québec G1V 0A6, Canada; Groupe de recherche interuniversitaire en limnologie (GRIL), Montreal, Québec H3C 3J7, Canada; Takuvik Joint International Laboratory, Université Laval, Québec, Québec G1V 0A6, Canada; Département de chimie, Université Laval, Québec, Québec G1V 0A6, Canada; Centre d’études nordiques (CEN), Université Laval, Québec, Québec G1V 0A6, Canada; Takuvik Joint International Laboratory, Université Laval, Québec, Québec G1V 0A6, Canada; Institut de biologie intégrative et des systèmes (IBIS), Université Laval, Québec, Québec G1V 0A6, Canada; Département de biologie, Université Laval, Québec, Québec G1V 0A6, Canada; Centre d’études nordiques (CEN), Université Laval, Québec, Québec G1V 0A6, Canada; Takuvik Joint International Laboratory, Université Laval, Québec, Québec G1V 0A6, Canada; Pacific Biosciences Research Center, University of Hawaiʻi at Mānoa, Honolulu, HI 96822, United States; Département des sciences fondamentales, Université du Québec à Chicoutimi, Chicoutimi, Québec G7H 2B1, Canada; Centre d’études nordiques (CEN), Université Laval, Québec, Québec G1V 0A6, Canada; Groupe de recherche interuniversitaire en limnologie (GRIL), Montreal, Québec H3C 3J7, Canada; Institut de biologie intégrative et des systèmes (IBIS), Université Laval, Québec, Québec G1V 0A6, Canada

**Keywords:** 16S rRNA gene, Arctic, biogeography, microbiomes, resilience, species sorting

## Abstract

Climate warming is likely to increase the physical connectivity of ecosystems with their surroundings. For Arctic lakes, increasing meltwater and precipitation may enhance the inputs of nutrients, organic matter and microorganisms from their catchments, and the increasingly ice-free, open-water conditions of the Arctic Ocean may favor increased inputs of marine aerosols, including microbiota. This study therefore aimed to determine how changing connectivity to terrestrial and marine habitats may affect the dispersal, sorting, and establishment of bacterial communities in a coastal High Arctic lake. Three habitats in this model system were sampled for ice, water, and snow: the lake, inflowing water tracks over permafrost soils, and an adjacent ice-dammed bay connected to the Arctic Ocean. Lake water chemistry confirmed the hydrological connection between the lake and terrestrial habitats, with the lake fed by terrestrial carbon sources via snow and groundwater run-off. Sequencing of 16S rDNA and rRNA showed evidence of a small marine and terrestrial influence on the lake, but few bacterial phylotypes were common to all three connected habitats. These results imply ongoing strong environmental filtering by habitat type, despite the apparent and potentially rising connectivity, and provide an example of bacterial resilience in a region of rapid climate change.

## Teaser

The microbiome of a High Arctic lake shows ecological resilience and divergence from terrestrial and marine communities, despite elevated hydrological connectivity.

## Introduction

In Arctic landscapes, the summer melting of snow combined with thawing of the permafrost active layer leads to increased hydrological connectivity from land to freshwaters. This connection results in the transport of terrestrial organic matter, nutrients, and microorganisms to downstream lakes (Crump et al. 2012, Dubnick et al. 2017, Comte et al. 2018, Kohler et al. 2020, Vrbická et al. 2022). During the melting season and over daily thaw cycles (Paquette et al. 2018), inputs to lakes from these terrestrial sources are likely to increase due to increased water flow and connectivity (Crump et al. 2003, Cavaco et al. 2019).

Arctic lakes are covered in ice and surrounded by snow-covered catchments for much of the year. These snow and ice surfaces act as receptors for aerosols, accumulating these materials and then releasing them into lake inflows or directly into the lake water during the annual summer thaw. Aerosols contain many types of materials including salts (Salter et al. 2016), pollen, proteins, spores, algae, bacteria, and viruses (Jaenicke 2005). In coastal regions, aerosols may also contain marine organisms, and represent a transport pathway for microbes from the sea to land. These marine microbiota include spore-forming taxa, such as *Firmicutes* (Bottos et al. 2014) and *Pseudomonadota* (Bottos et al. 2008, Cuthbertson et al. 2017, Malard et al. 2022). Summer melting of Arctic sea ice and open seas allow microbes to be transferred by wind and waves from the ocean to the atmosphere (King-Miaow et al. 2019), thereby providing a potentially large marine source of microbiota to coastal lakes.

Microorganisms are primarily passive dispersers (Martiny et al. 2006, Besemer et al. 2013), and dispersal processes can therefore be a major driving force for microbial community structure, diversity, and productivity. With ongoing climate change in the Arctic, the transport of cells and other materials from terrestrial runoff and atmospheric sources are both likely to increase. The supply of resources as well as community interactions within lake ecosystems may be transformed as new connections with microbial, organic carbon, and nutrient sources occur more frequently (Rillig et al. 2015). Arctic lakes are characterized by low nutrient levels (oligotrophy), prolonged ice cover that isolates them from the surrounding landscape, and simplified food webs (Vincent et al. 2008). Consequently, any novel inputs could potentially alter the chemical composition, biological community structure, and overall productivity of these polar ecosystems. Within-lake processes may amplify or mitigate microbial community responses to increased connectivity, and microbes newly introduced from terrestrial or marine sources may persist in the lake habitat, or may struggle to compete with established local species, leading to their failure to colonize and grow (Nemergut et al. 2013). Factors such as input volume, community stability, and local environmental conditions can affect these interactions and colonization success, while frequent mixing events may enable the coexistence of highly diverse communities (Rillig et al. 2015). Inflows from terrestrial habitats can influence lake microbial diversity in contrasting ways. A study of Lake Hazen, the world’s deepest High Arctic Lake, found a decrease in taxonomic and functional diversity of microbial communities following seasonal runoff (Colby et al. 2020), contrasting with observations from an Alaskan glacier-fed lake and another Arctic lake that indicated increased diversity (Choudhari et al. 2014, Comte et al. 2018).

Atmospheric connectivity and the influence of marine environments on freshwater microbial communities are still poorly understood. Some studies suggest that the deposition of airborne microbes to coastal lakes contributes to the seeding of new organisms, promoting changes in existing communities (Bowers et al. 2012, King-Miaow et al. 2019), and altering interactions between terrestrial and freshwater organisms. This issue is particularly relevant to northern high-latitude regions. As Arctic Ocean ice cover decreases (Crawford et al. 2021), there are likely to be enhanced interactions between marine and land-based ecosystems, but the implications for freshwater microbial communities remain little explored.

In this context, Ward Hunt Lake (WHL) located at the northern coast of High Arctic Canada provides a model system to evaluate the nature and extent of microbial dispersal and habitat selection, and at a time of rapid climate warming that is likely to strengthen the physical coupling across the ocean–land–lake continuum. The lake is located in the coastal margin of the Last Ice Area (LIA), which accumulates some of the oldest and thickest sea ice of the Arctic Ocean and is considered to be a refuge for marine ice-dependent species in the face of global warming (Newton et al. 2021). This persistent thick ice maintains the cold temperatures and polar desert regime of the coastal terrestrial environments of this region, however the coastal ocean has lost much of its landfast multiyear sea ice in recent years (Vincent and Mueller 2020) and is now experiencing periods of ice-free open water in summer (Klanten et al. 2024). Consistent with the trend of Arctic amplication of global warming, the island has recently experienced record extremes in melting degree days (Bégin et al. 2021a). As the northermost lake in Canada, WHL is at the forefront of climate change and therefore uniquely situated to evaluate the response of polar freshwater microbial communities to climate warming and associated changes in connectivity.

WHL is at an early stage of responses to climate change, but has already shown evidence of large impacts. Despite its historically thick and perennial ice-cover, it has experienced complete ice-off events in recent years (Paquette et al. 2015), leading to pronounced changes in water column structure and other limnological properties (Bégin et al. 2021b). The lake is supplied by snow-fed water tracks over permafrost soils (Paquette et al. 2017), and climate warming at this location is likely to deepen the active layer and enhance the mobilization of deeper soil particles to the lake. While lake ice protects WHL from much airborne deposition, the increased frequency of open water conditions both in the lake and nearby Arctic Ocean are likely to enhance the connectivity between these two environments. This increasing atmospheric and hydrological connectivity therefore made it an opportune time to determine whether there are already discernable effects on microbial ecology, specifically bacterial community structure, in this far northern lake ecosystem.

Here, our aim was to evaluate the extent of bacterial dispersal versus habitat filtering in a polar lake ecosystem experiencing the onset of climate warming. Specifically, we examined biogeochemical signatures of connectivity, other limnological properties, and the bacterial community structure and diversity of WHL, a natural laboratory for ecosystem studies that receives inflows from melting snow over permafrost soils and that lies only a few hundred meters away from a coastal embayment of the Arctic Ocean. We hypothesized that: (1) freshwater, terrestrial, and marine habitats have unique assemblages of microorganisms driven by local environmental conditions; (2) the lake habitat shares more bacterial and biogeochemical features with terrestrial rather than marine habitats due to greater connectivity with its land-based catchment; and (3) bacterial divergence among habitats is driven by the availability and origin of organic matter and nutrients. We evaluated the terrestrial input of organic carbon into the lake by spectrofluorometric analysis, and used 16S rRNA gene V3–V4 amplicon sequencing to compare the bacterial communities of the lake with those in surrounding habitats (marine and terrestrial).

## Methods

### Study site

**Figure 1.**
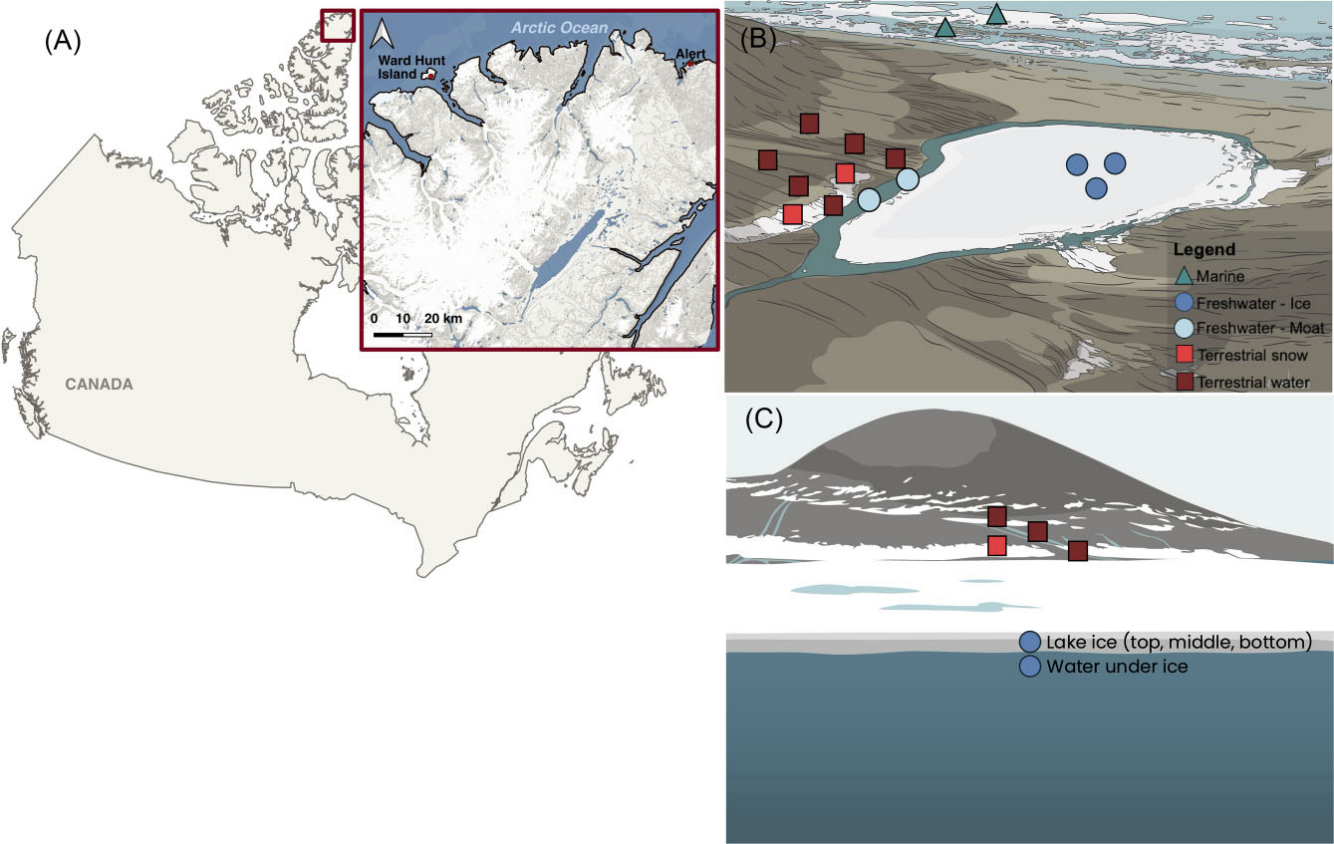
Sampling location on the northern coast of Ellesmere Island. (A) Map of WHI location. (B) WHL and sampling points from the freshwater lake ice and water under ice through three bore holes one meter apart (circle), freshwater lake moat (circle), surrounding terrestrial (square), and marine habitats (triangle). (C) Side view from WHL with lake sampling profile and terrestrial sampling points.

Sampling of Ward Hunt Island (WHI; 83° 06′ N, 74° 10′ W), Nunavut, Canada, was done in July 2022 (Fig. 1). The northern coast of WHI is bordered by a proglacial bay (Quttinirpaaq Lagoon, about 600 m north of WHL), a narrow band of water separating the island from the Ward Hunt Ice Rise, a major cryospheric feature in the area (Braun et al. 2004). Since the partial collapse of the Ward Hunt Ice Shelf in 2008, this system is connected to the Arctic Ocean and has become brackish (Vincent et al. 2009), with a surface conductivity of 3.5 ± 0.8 mS/cm in 2022 (Hooper et al., unpublished work). Soils on WHI are mainly composed of limestone and igneous and volcanic rocks (Trettin 1991), and the surface layer is organic-rich and contains a mix of gravel and sand (Paquette et al. 2017). The area is a polar desert, with an average annual temperature of −17.3°C (CEN 2022), and mean snow precipitation (water equivalent) of 158 mm/year (at Alert, 170 km southeast of WHI) (Government of Canada 2023, Paquette et al. 2015). Further details about WHI and vicinity are given in Vincent et al. (2011).

WHL is located in the center of WHI, about 700 m inland from the proglacial bay connected to the Arctic Ocean. The lake is 26 m above sea level, has a surface area of 0.35 km^2^ (Paquette et al. 2017) and a maximum depth of 9.7 m (Paquette et al. 2015). WHI is a small island that in the past was embedded in the landfast Ward Hunt Ice Shelf. This thick ice broke up in 2011, and today the island is largely surrounded by first-year sea ice and increasing open water in summer (see Fig. 1 in Davesne et al. 2022). The lake drains southward into Disraeli Fiord via an alluvial channel outlet and is surrounded by terrestrial (snow and permafrost) and marine (Arctic Ocean, coastal bay, and ice shelf) features. WHL is oligotrophic with well-developed biofilms communities in both the shallow moat and deepest parts of the lake (Mohit et al. 2017). Rotifer communities have also been observed at the littoral margin of the lake and under the ice, but crustacean zooplankton are absent (Bégin et al. 2021a). The surrounding watershed includes cyanobacterial communities associated with snowpacks (Harding et al. 2011) and a network of water tracks that channel meltwater to the lake (Paquette et al. 2017). Historically, the lake has been covered by ice year-round, with thickness reaching up to 4.3 m in the 1950s (Hattersley-Smith 1963). This ice cover has experienced thinning and a reduced area in recent decades, culminating in ice-free summers in 2011, 2012 (Paquette et al. 2015), and 2016 (Bégin et al. 2021c). The disappearance of ice on WHL promotes mixing, which decreases average light availability by increasing turbidity in the water column, altering the lake by limiting primary production (Bégin et al. 2021c). The lake has maintained its ice cover over past years and has not been completely ice-free since 2016. Nevertheless, during the spring, an ice-free moat up to 10-m wide forms at the margin of the lake, promoting exchanges with its watershed and the atmosphere (Bégin et al. 2021a). As the lake ice cover is not attached to the bottom of the lake, there are exchanges between the under-ice water column and the moat. However, the lake’s substantial through flow toward the outlet restricts these exchanges.

### Sampling

#### Water sampling

Temperature, oxygen, chlorophyll-*a* (Chl-*a*), and conductivity in the water column were measured using a Ruskin RBR concerto^3^ profiler (RBR, Ottawa, Canada) in WHL through three holes bored 1 m apart in the ice cover. Lake water was collected under ice (0–100 cm) from the stratified water column using a 7-l Limnos bottle (Limnos.py, Komorów, Poland) as well as from the moat by grab sample using a clean 1 l Nalgene^©^ bottle. Water was also collected from the surface waters of proglacial Quttinirpaaq Lagoon in triplicate. Lake and ocean water was stored in 4 l high density polyethylene (HDPE) cubitainers previously washed with 2% (v/v) Contrad™ (DeconLabs, King of Prussia, PA, USA), 3.7% hydrochloric acid (HCL) (Sigma-Aldrich, St. Louis, MO, USA) and MilliQ water and rinsed with site water. For water tracks, 150 ml of water was collected with sterile Luer Lock 60 ml syringe (Fisher Scientific, Waltham, MA, USA) and transferred into sterile Falcon™ tubes (Fisher Scientific).

#### Ice and snow sampling

Ice cores were collected in triplicate, one meter apart, using a Mark II ice corer (Kovacs Enterprise, Roserburg, OR, USA) from lake ice, the proglacial bay, and sea ice, for a total of nine cores. At the time of sampling in July 2022, ice covered the majority of WHL and was 155 cm thick. The ice was multiyear (it had not entirely disappeared since 2016) and remained for the rest of the season. Lake ice cores were collected in full and were cut using an ethanol-washed saw and ice pipe and stored in 5441 ml sterile Whirl-Paks^TM^ (Filtration Group, Austin, TX, USA). Proglacial bay and sea ice cores were collected from the surface (~1 m long) and sliced at 50 cm (0–50 cm and 50–100 cm). During ice core handling, precautions were taken to avoid contamination (ethanol-washed tools, working on autoclaved and ethanol-washed plastic surfaces, and ethanol-washed gloves) (Christner et al. 2005). Cores were kept on the field in open coolers covered with opaque black bags to protect them from solar radiation as they melted over 24–72 h prior to subsampling and filtering. Snow was collected using an ethanol-washed HDPE shovel into 5441 ml sterile Whirl-Pak^TM^ bags, in which it melted in the dark at 4°C until subsampling and processing.

### Sample processing

#### Nutrient and water chemistry analyses

Samples for total phosphorus (TP) and total nitrogen (TN) were obtained in duplicate from lake water, ice and snow meltwater and stored in 50 ml Falcon™ tubes. Water samples for dissolved organic carbon (DOC) were filtered through 0.2 µm Acrodisc™ filters (Pall Corporation, New York, NY, USA) and stored in amber glass bottles until analysis. Samples for the determination of colored dissolved organic matter (CDOM) were prefiltered through 47 mm GF/F filters (Whatman, Maidstone, UK) and stored in amber glass bottles previously cleaned with acid (0.1 M HCl) and burnt (6 h at 450°C). TP, TN, DOC, and CDOM samples were kept in the dark at 4°C until analysis. The potential origins of the dissolved organic matter (DOM) were determined via spectrofluorometric quantification (Cary Eclipse, Agilent, Santa Clara, CA, USA) combined with PARAFAC parallel factor analyses. The absorption coefficient at 320 nm (*a*_320_), specific ultraviolet absorbance (SUVA_254_), and spectral slope at 289 nm (S_289_) were calculated from the excitation–emission matrices (EEMs). These variables were used, respectively, as indices for DOM color (*a*_320_), aromatic terrestrial DOM (Weishaar et al. 2003), and autochthonous DOM (Loiselle et al. 2009, Wauthy et al. 2018). A PARAFAC model was run using *stardom* (V1.1.28) (Pucher et al. 2019) on EEMs processed using *eemR* (V1.0.1) (Massicotte 2017) packages in R (V4.3.1) (R Core Team 2021). Slip-half analysis identified a three component model as the best approach (*R*^2^ = 0.88), from which sources of DOM were identified using the Open Fluor online library (Murphy et al. 2014).

#### Pigment extraction and quantification

Chl-*a* was used as an indicator of algal biomass. For each sampling site, two or three replicates of 150 ml water were subsampled from lake and meltwater and filtered through 25 mm GF/F glass microfiber filters (Whatman). The filters were stored at −20°C in the field and at −80°C on return to Université du Québec à Chicoutimi (UQAC), where Chl-*a* extraction was performed within 4 months of sampling using hot ethanol (95% in a hot bath at 70°C for 5 min) prior to pigment quantification, performed using a Cary Eclipse spectrofluorometer and a calibration curve obtained from spectrophotometer measurements. Measurements were taken before and after acidification (3.7% HCl) to correct for phaeopigments, as described in Nusch (1980).

#### Cell and viral particle counts

Bacteria, microbial eukaryotes, and viral particles were counted by flow cytometry to provide information on the abundance of different major functional groups. For viruses, 980 µl of lake or meltwater was subsampled and fixed in a 2-ml cryovial, preloaded with 20 µl of 25% grade I glutaraldehyde (Sigma-Aldrich). For the cellular fraction, 3840 µl of water was added to a 5-ml cryovial preloaded with 160 µl of 25% glutaraldehyde (final concentration 1%). Both cellular and viral cytometry samples were made in duplicate and incubated for at least 15 min in the dark before being stored at −20°C. These samples were analysed at the Institut des sciences de la mer de Rimouski (ISMER, Rimouski, Canada), as described in Brussaard et al. (2010).

#### Stable water isotopes

Stable water isotopes were analysed to assess the hydrological connectivity between habitats. For each site, duplicate lake or meltwater subsamples were collected for isotopic analyses of δ^2^H and δ^18^O, to identify the origin of the water transported into the lake. For each isotope, water was collected with no headspace into white 30 ml HDPE Nalgene™ bottles and stored at 4°C prior to analysis by the Geotop analysis center (Université du Québec à Montréal, Montréal, Canada). Isotopic values were used in a linear regression to describe the exchange of water between habitats based on the global meteoric water line (GMWL; δ^2^H = 8 × δ^18^O + 10‰) (Craig 1961, Gat 1996) and the local meteoric water line (LMWL; δ^2^H = 7.62 × δ^18^O + 1.64‰) (Lacelle 2011). Deuterium excess (d-excess) was calculated as a proxy for evaporation (Dansgaard 1964) based on the hydrogen deuterium (δ^2^H) and oxygen isotope (δ^18^O) values using the following equation: $d - \textit{excess} = \;{\delta ^2}H - 8*{\delta ^{18}}O$.

#### Nucleic acid extraction

For bacterial community analyses, samples were filtered in triplicate onto 0.22 µm Sterivex™ filters (Millipore, Burlington, MA, USA) using a Masterflex L/S peristaltic pump (Cole-Parmer, Vernon Hills, IL, USA) and Contrad- and HCl-washed tubing (as described above). For lake and meltwater (ice and snow) samples, a minimum of 1 l of water was filtered, while 150 ml was filtered for water tracks. Filters were air-dried, then fixed with 2 ml of RNAlater™ (Invitrogen, Waltham, MA, USA) and stored at −20°C until their transport to UQAC, where they were kept at −80°C until further processing. DNA and RNA were coextracted from Sterivex filters using the AllPrep DNA/RNA Mini extraction kit (QIAGEN, Hilden, Germany), following the protocol described in Cruaud et al. (2017). Following DNA and cDNA extraction, quality control of each sample was performed using a Qubit 4 Fluorometer (Invitrogen). Retroconversion (from RNA to cDNA), library preparation (for DNA and cDNA), and amplicon sequencing of the V3–V4 region of the 16S rRNA marker gene were performed at the Plateforme d’analyses génomiques at the Institut de biologie integrative et des systèmes (Université Laval, Québec, Canada). Sequencing primers used for both DNA and cDNA (from RNA) were forward 341F (CCTACGGGNGGCWGCAG) and reverse 805R (GACTACHVGGGTATCTAATCC) (Herlemann et al. 2011). Sequencing yielded 1 492 673 reads for DNA (average of 57 410 per sample) and 1 829 249 reads (average of 70 355 per sample) for cDNA.

### Data analysis

#### Water residence time

The water residence time (WRT) of WHL was estimated based on the equation of Ward and Elliot (1995):


\begin{eqnarray*}
WRT = \;\frac{V}{{CA \bullet P \bullet EF\;}},
\end{eqnarray*}

where *V* is the lake volume (m^3^), *CA* is the catchment area (m^2^), *P* is the annual precipitation (mm/year), and *EF* is the evaporation adjustment factor for the catchment (100 − $\frac{{\% \textit{loss}}}{{100}}$). WRT (y) was calculated for both the whole lake and the moat section only. For the lake inflows, the average catchment evaporation loss was assumed to be 50% while the moat inflows were assumed to be half the total received by WHL and the average moat depth was estimated as 0.5 m.

#### Sequence processing

Reads were processed in RStudio (2023.06.2+561). Primers and low-quality reads were trimmed and chimeras were removed in *dada2* (V1.28.0) (Callahan et al. 2016) using the following parameters: trimLeft = c(17,21), truncLen = c(280 260), maxN = 0, maxEE = c(2,2), and truncQ = 2. The number of reads per sample were tracked throughout sequence processing, and five DNA samples were removed from downstream analyses due to low sequence counts (<1000 reads). These excluded samples were: one bottom lake ice, two lake moat, and two marine samples (Table S1). Following sequence cleaning and processing, a total of 251 696 bacterial reads were retained from all DNA samples. From these, 102 013 sequences were recovered from the marine samples, 95 776 for freshwater and 53 897 for terrestrial. Taxonomy was assigned in *dada2* using assignTaxonomy() and addSpecies() with the SILVA v138.1 database (Quast et al. 2012). *Phyloseq* (V1.44.0) (McMurdie and Holmes 2013) was used for diversity analyses. A raw amplicon sequence variant (ASV) table was used for alpha diversity index calculations (species richness). For community analyses, low abundance taxa were removed (<5% across samples, leaving 784 ASVs), and abundance tables were standardized by total square-root method (Hellinger with decostand() in *vegan* (V2.6–4) (Oksanen 2017) and log-transformed.

#### Statistical analyses

Difference between DNA and RNA bacterial communities was tested on log- and Hellinger-transformed data with adonis(), a PERMANOVA variance-based test in *vegan*. To ensure that the test was not biased by within-group variability, a betadisper() test followed, also in *vegan*. To visualize the 10 most abundant phyla, tax_glom() was used in *phyloseq*. Differences in alpha diversity between DNA and RNA was determined by performing a T-test on normally distributed data for Observed and Shannon indices. Prior to analysis, we confirmed species saturation using rarefaction curves (Fig. S1). To compare community composition of samples, a nonmetric multidimensional scaling (nMDS) was performed on the log- and Hellinger-transformed data using a Bray–Curtis dissimilarity matrix. Difference among habitats was again tested using adonis(), followed by a betadisper() to determine if there was within-group variability. A Venn diagram was computed showing unique and shared ASV across habitats and plotted using *ggvenn* (V0.1.10) (Gao et al. 2021) on raw data (including singletons). Phylogenetic distance among bacterial communities from different habitats was calculated using *ape* (V5.7–1) (Paradis et al. 2004) based on a Unifrac weighted distance matrix. Each matrix was then compared among groups based on their phylogenetic dissimilarity by performing an ANOSIM test using *vegan*.

Prior to environmental data analysis, normality of data distribution was assessed by performing a Shapiro test in R. Physiochemical values among habitat were compared using ANOVA followed by Tukey multiple comparison *post hoc* test using the *vegan* package. Furthermore, to determine the contribution of physicochemical parameters as explanatory variables to bacterial patterns across the habitats, a bioenv() analysis was performed in *vegan* using a Bray–Curtis dissimilarity matrix of community composition compared to a transformed and normalized environmental data matrix. Spearman correlations among physicochemical parameters were tested using the cor() function in R before performing the test to remove the covariables, revealing that Chl-*a* was highly correlated to SUVA_254_ (84%), TP (89%), dissolved inorganic carbon (DIC; 89%), and component C3 (86%). In addition, component C2 was highly correlated to S_289_ (84%) and SUVA_254_ was highly correlated to *a*_320_ (84%). Tested variables for the BIOENV model included : Chl-*a*, SUVA_254_, PARAFAC DOM components C1, C2, and C3, DIC, and S_289_. All statistical analyses were performed using *vegan*.

## Results

### Limnological and environmental conditions

The WHL water column was stratified with respect to temperature, conductivity, and dissolved oxygen (Fig. S2). Water temperature increased with depth and stabilized around 3 m, and then slightly decreased to 8 m. Specific conductivity was higher (0.254 mS/cm) in the bottom of the lake than in the upper 8 m of the water column (0.125 ± 0.06 mS/cm). Dissolved oxygen was highest at 6 m (123.4 µmol/l; 30% air equilibrium) but dropped to hypoxic values at the bottom of the water column (22.7 µmol/l at 8 m; 5% air equilibrium). WRT for WHL was calculated as 10 years for the whole lake, but only 10 days for the moat assuming minimal exchange with offshore waters (Table S2).

Chl-*a* concentrations were highly variable depending on the type of sample (freshwater, ice, snow, and marine) (*P* < .05). Chl-*a* in lake water sampled directly under ice (5.4 ± 2.4 µg/l) was 67-fold higher than in lake ice (0.08 ± 0.04 µg/l), and four times higher than the concentration measured in the marine habitat (both lagoon and sea ice) (1.2 ± 1.0 µg/l) (*P* < .001). However, Chl-*a* concentration in lake ice was similar to marine measurements in water and sea ice (*P* > .05) (Table S3).

SUVA_254_ and *a_320_* values were greater in terrestrial snow samples than in freshwater and marine habitats (Fig. S3A and B). In the lake water sampled directly under ice, *a_320_* values were similar to both terrestrial snow and freshwater ice. S_289_ values increased along the terrestrial–freshwater–marine continuum, with values significantly greater in marine than in terrestrial habitats (Fig. S3C). Nutrient concentrations varied among freshwater samples (Table S3). TP concentrations were two-fold higher in the water under-ice than in lake ice and marine samples (8.0 µg/l vs 15.7 µg/l) (*P* < .001). The same trend was observed for DIC where values from water under-ice were five-fold greater than in lake ice (1.4 µg/l vs 5.4 µg/l) and sea ice (1.98 µg/l) (*P* < .005). DOC concentrations were significantly lower in water under-ice than in ice samples (*P* < .005). Finally, TN concentrations were similar for ice and water from both the lake and marine environment (*P* > .05).

Flow cytometry counts showed that the number of bacteria per volume was lower in WHL (lake ice and water under-ice) than in marine and terrestrial habitats (*F* = 8.707, *P* < .005) (Table S4). The number of viral particles was not significantly different across habitats (*F* = 1.942, *P* > .05).

**Figure 2.**
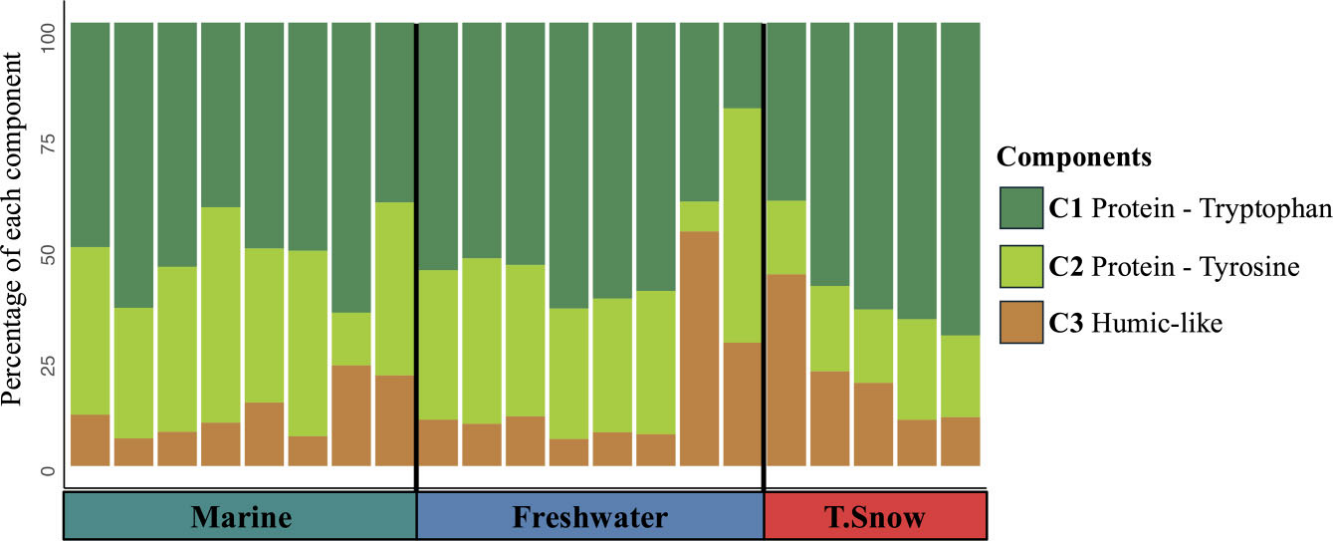
PARAFAC components for sampling locations based on the marine (lagoon and sea ice), freshwater (WHL ice and water under ice) and terrestrial (snow) habitat on WHI in 2022 expressed in percentage, based on Raman units.

PARAFAC analysis revealed that in general, autochthonous bacterial material or protein like components (C1–C2) represented the most abundant form of organic matter in all habitats, accounting for 84 ± 17% of carbon (Fig. 2). Less than 20% of humic-like organic matter was also present in all samples but represent a lower source of organic matter (C3). However, lake water collected under the ice and some of the snow samples had >20% humic-like organic matter, with values reaching up to 53% (Fig. 2).

**Figure 3.**
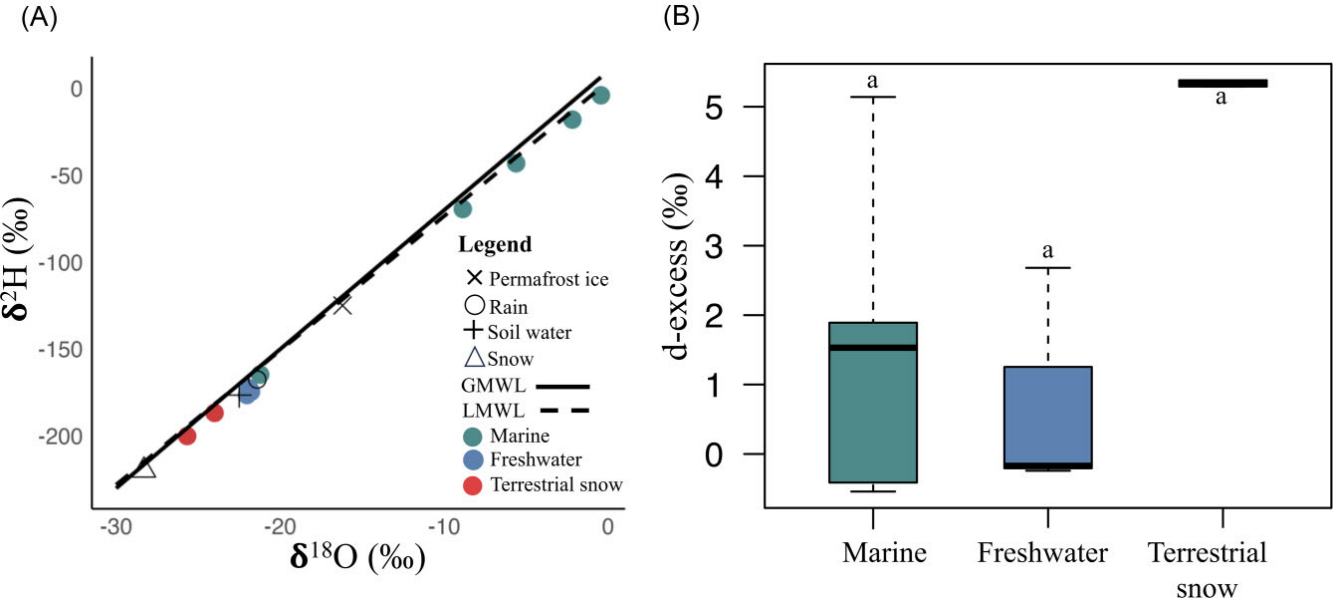
(A) δ^18^O and δ^2^H isotopic composition of marine (light blue), freshwater (dark blue), and terrestrial (brown) samples of WHI with reference to main sources of water. The black continuous line represents the GMWL (δ^2^H = 8 × δ^18^O + 10‰) and the dotted line represents the LMWL (δ^2^H = 7.62 × δ^18^O + 1.6‰). (B) d-excess for the marine (*n* = 5), freshwater (*n* = 3), and terrestrial snow (*n* = 2) samples. Letters indicate no significant differences among groups based on ANOVA (*P* > .05) and *post hoc* Tukey’s test.

Marine habitats had the heaviest isotopic ratios followed by freshwater (ice and lake water under-ice) and terrestrial snow (Fig. 3A, Table S5). Comparing values to the GMWL indicated that WHL receives contributions from both precipitation and ground water. Measurement of d-excess, as a proxy for evaporation, indicated no significant difference among marine, freshwater, and terrestrial snow samples (ANOVA *P* > .05) and little evaporation (Fig. 3B).

### Bacterial communities from DNA and cDNA (RNA)

**Figure 4.**
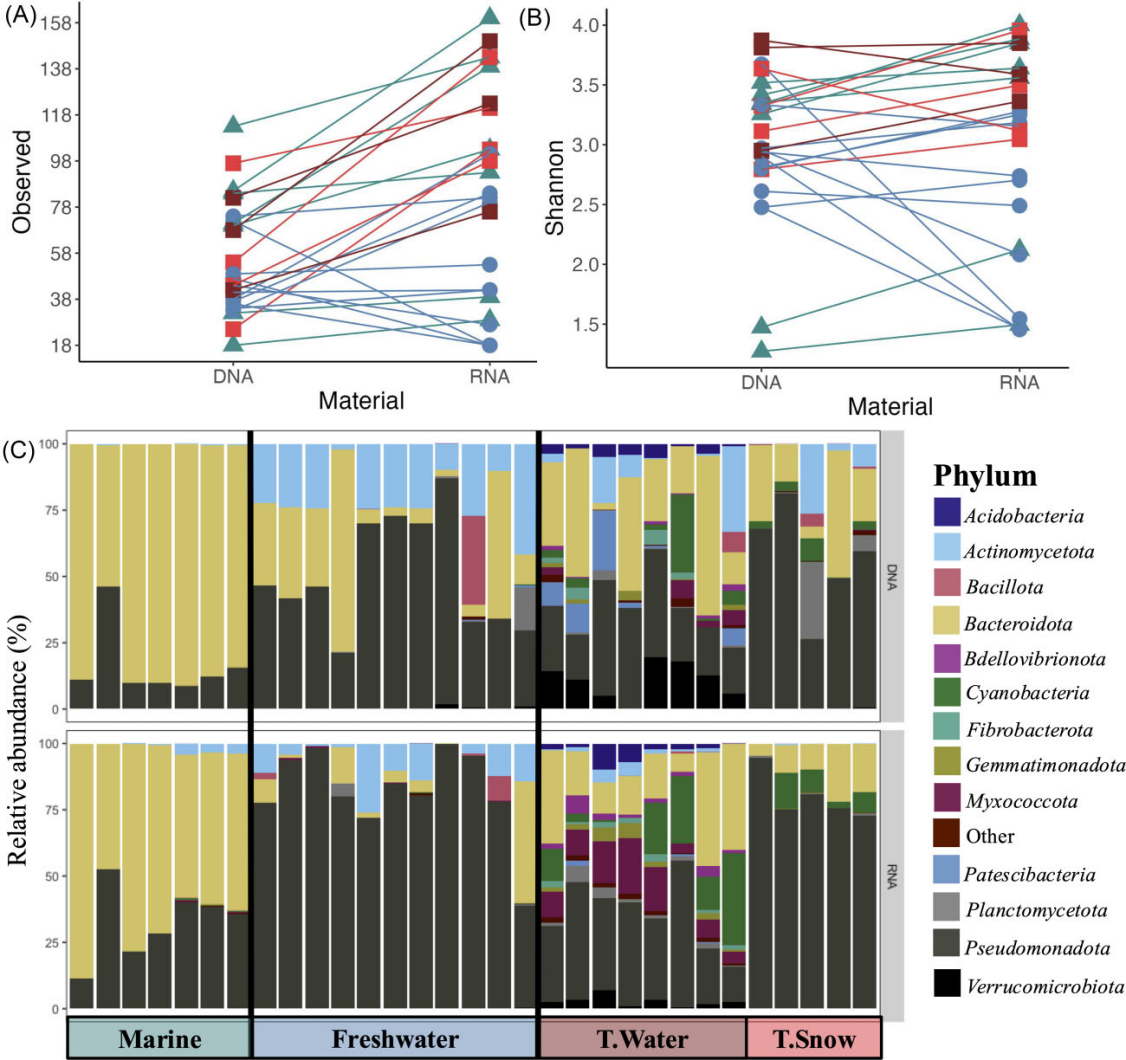
(A) Observed species and (B) Shannon alpha diversity indices compared between paired DNA and cDNA (RNA) datasets. Colors and shapes represent the origin of the samples shown in C, lines connect paired DNA and cDNA samples. No significant difference in diversity was observed for both richness indices (ANOVA *P* > .05) (C) Relative abundance of bacterial phyla based on DNA and cDNA (RNA) (normalized and transformed data, Hellinger and log), for samples from marine (lagoon and sea ice), freshwater (lake water under the ice and ice lake), terrestrial water (water tracks), and terrestrial snow habitats on WHI. Category “Other” includes low abundance phyla (<5% combined).

A total of 867 022 bacterial reads were recovered from DNA and cDNA sequencing following data cleaning and processing (Table S1). From all the sequences recovered 1518 ASVs were identified prior to singletons removal. Bacterial community assemblages were different among DNA and RNA (PERMANOVA *F* = 1.696, *P* < .05) and RNA samples showed greater Observed richness (ANOVA *P* < .05, 56 ± 24 in DNA and 83 ± 45 in RNA). However, Shannon indices were not different between DNA and RNA samples (*t*-test, *P* > .05, 3.00 ± 0.62 for DNA and 2.97 ± 0.83) (Fig. 4A and B).

The most abundant phyla in DNA samples were also prevalent in cDNA reads. There was a dominance of the phyla *Pseudomonadota* and *Bacteroidota* in both nucleic acid datasets (Fig. 4C). This trend is also seen when comparing results based on the type of habitat (marine, freshwater, or terrestrial). In general, the same phyla were consistently detected across habitats but in different proportions, especially for the marine and freshwater habitats, which showed the greatest pairwise dissimilarities.

### Bacterial dissimilarity among habitats

**Figure 5.**
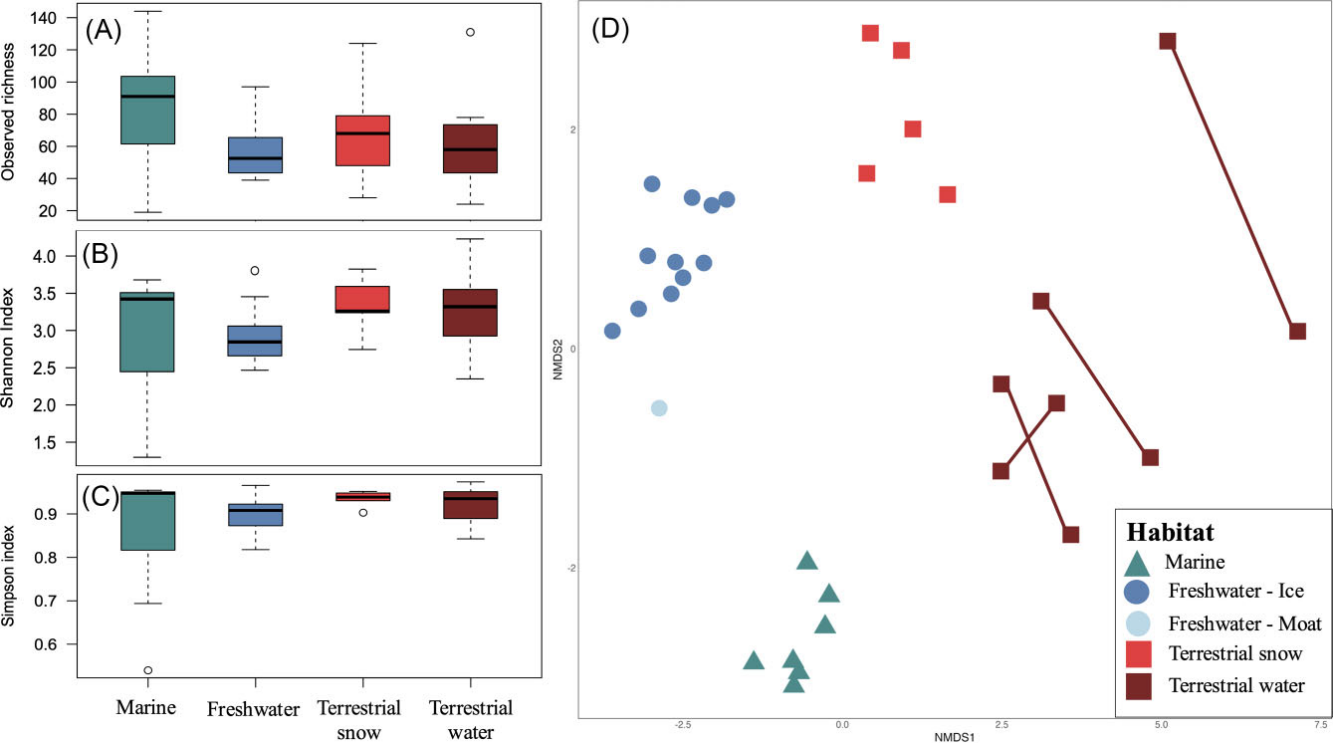
Taxonomic richness of bacterial DNA measured with (A) Observed, (B) Shannon, and (C) Simpson diversity indices based on habitat. Number of samples for each habitat was: marine *n* = 7, freshwater *n* = 11, freshwater moat *n* = 1, terrestrial snow *n* = 5, and terrestrial water *n* = 8. No significant difference was found in species richness between habitats for any index (*P* > .05). Boxes represent interquartile distribution, thick black line is the median, dotted lines are for the distribution of the data’s first and third quartiles and empty dots are for outliers. (D) Dissimilarity among microbial communities (based on Hellinger and log transformed abundance and Bray–Curtis dissimilarity matrix) between marine, freshwater, and terrestrial habitats on WHI visualized in a nonmetric multidimensional scale. Differences between habitats were tested using a PERMANOVA (*F* = 3.579, *R*^2^ = 28%, *P* = .001); and within habitats with betadisper (*F* = 2.326, *P* = .104). Lines connect replicates for terrestrial water samples due to their greater dissimilarity.

Across all DNA samples, Observed species, Shannon diversity index, and Simpson equitability were not significantly different (*P* > .05) across marine, freshwater, and terrestrial habitats (Fig. 5A–C).

Although there were no differences in richness values, the bacterial communities differed significantly in terms of their assemblages among the three types of habitats (PERMANOVA, *P* < .005) (Fig. 5D), and habitat type explained 28% of variation in community composition. Each habitat clustered with samples from the same origin along both principal axes. Freshwater samples (including lake ice and water from directly under the ice and the moat) grouped together, despite the moat differing slightly from the rest of the lake samples. Finally, water track samples from the terrestrial habitat were the most heterogenous, with replicates showing more variation (Fig. 5D).

The most abundant phyla were similar among all the sampled habitats (Fig. 4C, top panel). Marine communities (based on DNA) were mainly represented by members of *Bacteroidota* (83 ± 13%) and *Pseudomonadota* (16 ± 13%), with all other phyla accounting for <1% of reads. Freshwater communities were composed of three major phyla: *Pseudomonadota* (48 ± 21%), *Bacteroidota* (28 ± 27%), and *Actinomycetota* (20 ± 11%), while the remaining phyla accounted for the remaining 5% of reads. In general, terrestrial samples (snow and water) had more even communities and were not dominated by one or two phyla as were marine and freshwater samples (Figs 4C and 5C). Terrestrial samples also contained many phyla that were not prevalent in freshwater and marine habitats (*Acidobacteria, Myxococcota, Verrucomicrobiota*, and *Cyanobacteria*). *Pseudomonadota* and *Bacteroidota* represented more than half of the terrestrial water communities (respectively of 27 ± 11% and 30 ± 18%), but a large proportion of the community (43%) belonged to low-abundance phyla (<10%) (Fig. 4C, top panel). Bacterial communities from terrestrial snow were mainly composed of members of the *Pseudomonadota* (57 ± 20%), *Bacteroidota* (23 ± 16%), and *Actinomycetota* (7 ± 10%) phyla, with the rest being composed of diverse low-abundance phyla (13%).

**Figure 6.**
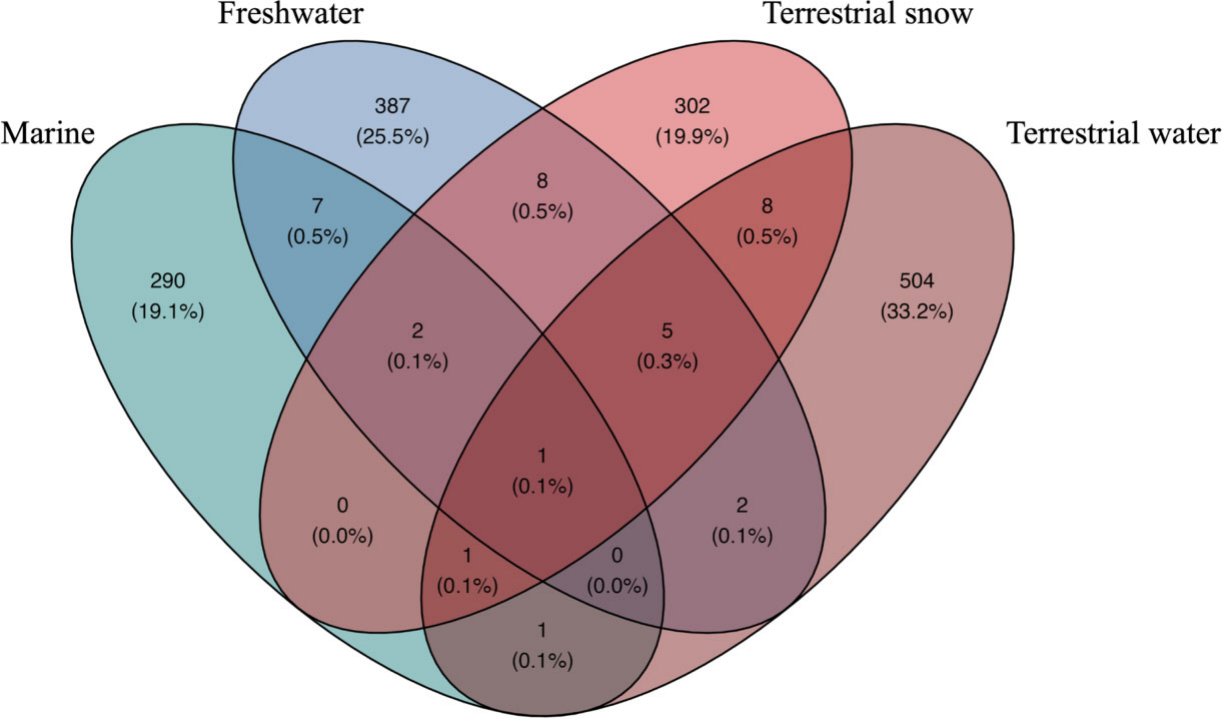
Unique and shared ASVs (including singletons) based on bacterial DNA among the different habitats on WHI, with their overall proportion reported in parentheses.

Bacterial community composition based on DNA also differed markedly among habitats (Figs 4 and 5). This difference was also underlined by the core community analysis, where 98% of ASVs are not shared, meaning only 2% of ASVs are shared between two or more habitats. Terrestrial water was the most unique habitat (504 unique ASVs) followed by freshwater (387), terrestrial snow (302), and finally the marine habitat (290) (Fig. 6). Among all ASVs, only one was shared among all habitats (unknown member of the genus *Prauserella*), meaning that only 0.1% of ASVs formed the WHI “core” community. Marine and terrestrial (snow and water) shared few ASVs (only two ASVs: unknown member of the genus *Polaromonas* and a *Chloroplast* sequence) while freshwater and terrestrial (snow and water) samples shared somewhat more (15 ASVs). Finally, terrestrial snow and terrestrial water shared fewer ASVs than with freshwater samples, with only eight shared ASVs between these two terrestrial habitats (Fig. 6).

**Figure 7.**
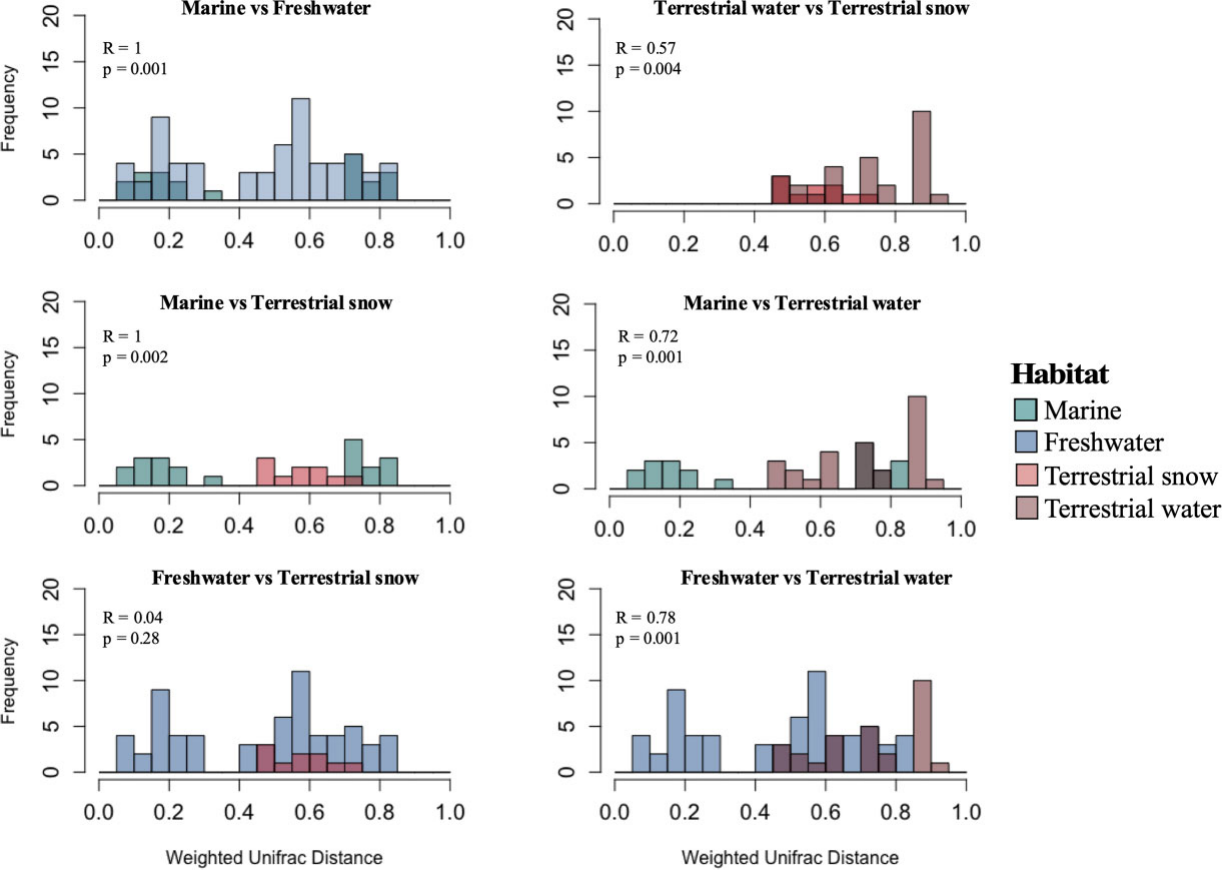
Histograms of weighted Unifrac distances based on bacterial DNA calculated between samples from different habitats: marine (cyan blue), freshwater (dark blue), terrestrial snow (red), and terrestrial water (brown) with associated ANOSIM *R*- and *P*-values.

Weighted Unifrac distances revealed bacterial communities in WHL and its surrounding habitats had distinct phylogenies. Bacterial communities between marine and freshwater samples were phylogenetically distinct (ANOSIM *R* = 1, *P* < .001), and marine and terrestrial habitats also showed little overlap (Fig. 7), with distinct phylogenies for both terrestrial snow and terrestrial water samples (ANOSIM *R* = 1 and 0.72, *P* < .001). Freshwater and terrestrial water samples were also highly dissimilar (*R* = 0.78, *P* < .001). However, freshwater and terrestrial snow bacterial communities were similar (ANOSIM *R* = 0.04, *P* = .28). Finally, bacterial communities between terrestrial water and terrestrial snow samples had distinct phylogenies (ANOSIM *R* = 0.57, *P* < .004) (Fig. 7).

### Drivers of bacterial dissimilarity in the WHL watershed

Bacterial communities in each habitat differed in their relationships with environmental variables (Table S6). Marine bacterial community structure was highly correlated with Chl-*a* concentration (Spearman *ρ* = 0.8), as well as to SUVA_254_, DIC, and the PARAFAC C1 and C2 fractions (*ρ* = 0.7), while lake communities were correlated with C1 (*ρ* = 0.7). However, other models for freshwater samples also identified Chl-*a* and S_289_ as explanatory variables (*ρ* = 0.6). Finally, bacterial community structure of terrestrial snow was correlated with SUVA_254_ and component C2 (*ρ* = 0.3).

## Discussion

The aim of this study was to evaluate the extent of bacterial dispersal versus habitat filtering in a polar coastal lake ecosystem. Our observations took place at an early stage of Arctic amplication of climate change in this extreme northern location, during which the ecosystem has been recently opened to atmospheric inputs as the result of lake ice loss from climate warming (Paquette et al. 2015). This may be accompanied by increased exposure to marine influences associated with recent sea ice loss in the coastal margin of the LIA (Vincent and Mueller 2020), as well as by potential changes in runoff conditions in the warming climate.

WHL showed clear evidence of hydrological connectivity with its surrounding watershed in terms of water chemistry and stable isotope signatures, and by the inferred presence of terrestrial-derived organic matter in the lake. Despite these hydrological connections, however, there were pronounced differences in bacterial community structure among habitats. The results show an overriding effect of local environmental pressures and selection processes, which have allowed beta diversity to be maintained during this early phase of ongoing environmental change.

### Hydrological connectivity in the WHL watershed

The water chemistry in WHL reflected the combined inputs of snowmelt and soil leaching, underscoring the hydrological connectivity to the terrestrial habitat. Terrestrial snow and water tracks were rich in organic compounds and fluorescent material as seen by their elevated SUVA_254_ and *a_320_* values. Greater concentrations of nutrients in surface lake water suggest leaching of nutrients from soils and ground water to the lake, as has been observed in the western Canadian High Arctic (Ayala-Borda et al. 2021) and in streams of the McMurdo Dry Valleys, Antarctica (McKnight et al. 1999). Slightly greater TP concentrations in the water directly below lake ice could result from the geology of WHI, which is rich in carbonate soils from sedimentary and volcanic rocks (Trettin 1991, Paquette et al. 2017). Therefore, terrestrial phosphorus inputs from water tracks and surface streams that flow into the lake immediately beneath the lake ice, as well as solute exclusion during freeze-up (Imbeau et al. 2021) may support greater primary production, and explain the higher Chl-*a* concentrations in water under the lake ice relative to lake ice. In addition to phosphorus enrichment, greater concentrations of Chl-*a* measured under the ice may be attributable to enhanced light availability immediately beneath the ice (Bégin et al. 2021b, Arrigo et al. 2012), below-ice intrusion of nutrient inputs from the watershed, and exclusion of particulate organic carbon and soluble phosphorus from the ice (Imbeau et al. 2021). Chl-*a* concentrations were greater in the water under-ice than in ice and snow, and were similar to values measured previously in WHL in both lake ice (Imbeau et al. 2021) and in the water column, consistent with oligotrophy (Bégin et al. 2021a).

PARAFAC analysis revealed that DOC content in water under-ice was influenced to various degrees (6%–50%) by humic-like compounds (Chen et al. 2017, D’Andrilli et al. 2019, Jeon et al. 2021) of allochthonous origin (C3) (Mohit et al. 2017). This C3 component was also abundant in terrestrial snow, which suggests that snow could be an important source of organic matter to the lake, as previously suggested by other studies performed on WHL (Bégin et al. 2021c, Comte et al. 2018). Snow acts as a receptor of organic material that is accumulated and trapped across the landscape, or can accompany precipitation throughout the year. Component 1 (C1), which represented on average 53% of the carbon source in samples, is likely from a protein-like tryptophan compound, as identified in many other aquatic systems (Murphy et al. 2008, DeFrancesco and Guéguen 2021). Component 2 (C2) also abundant in the samples (6%–52%) was from an autochthonous origin and was likely associated with protein-like tyrosine compounds (Chen et al. 2018, Retelletti Brogi et al. 2019, Derrien et al. 2020). In general, the ice samples contained low allochthonous inputs and were dominated by protein-like compounds from autochthonous bacterial activity, consistent with observations on WHL ice by Imbeau et al. (2021). Dominance of protein-like C1 and C2 in most of samples suggests recent biological production by microorganisms (Moran et al. 2000, Cammack et al. 2004), highlighting the central role of microbes in the short food web of WHL (Vincent 2018).

The isotopic composition of WHL waters indicated that the lake is mostly supplied by precipitation and ground water (runoff from snow, soil and water tracks) (Paquette et al. 2018). WHI is subject to an extreme polar desert climate (Vincent et al. 2011), and precipitation mainly occurs as snow, which then melts and fuels the runoff during summer. The main water sources in the lake identified through stable isotopes support the PARAFAC results and the gradient in SUVA_254_ and *a_320_*, and indicate a close hydrological connection between the lake and the watershed during the summer, with a strong signal of terrestrial DOM in the lake water under ice. WHL water chemistry is likely less impacted by marine inputs given the distance to the sea, and the protection of the lake from airborne transport by its multiyear ice cover that persists over most of the lake during summer and all of the lake through most of the year.

All three habitats on WHI (freshwater, marine, and terrestrial) were affected to a limited extent by evaporation as evidenced by the low deviation of values from the global and local meteoric water line and the positive values for the deuterium excess. These results are in line with a previous study conducted in the Canadian Arctic and the Greenland Sea stating that there is an increase in precipitation (Kopec et al. 2016) with current warming which leads to less evaporation. However, evaporation is highly variable among regions and locations, especially for freshwater, since it not only depends on temperature and precipitation but also on lake size, depth, and ice cover. A study of 49 water bodies in the Canadian High Arctic revealed both low and high evaporation rates for lakes on Ellesmere Island (Michelutti et al. 2022). The prolonged, often multiyear ice cover on WHL and its location in an extreme cold climate likely make it less prone to evaporation, increasing the role of inflowing runoff from the watershed in the lake water balance. Changes in Arctic precipitation, including a shift from snowfall to liquid rainfall, may shift the hydrological balance in WHL (Bintanja and Andry 2017), in addition to severely impacting the thickness and properties of lake ice (Brown and Duguay 2011) and enhancing surface inflows.

### Detected versus active bacterial communities

We used a combination of DNA and cDNA sequencing, to describe bacterial communities of WHL (DNA), but also to determine which fraction of the community is likely to be more active (cDNA). The bacterial community assemblages were consistently distinct between habitats for both sets of nucleic acid sequences. In general, phyla that were the most abundant and detected by DNA sequencing were also the ones that were the most potentially active (cDNA) at the moment of sampling, except for some phyla of the terrestrial habitat (*Actinomycetota, Planctomycetota*, and *Verrucomicrobiota*). *Bacteroidota* and *Pseudomonadota* were the two most abundant phyla among all our samples (both in DNA and cDNA), as in many other studies of Arctic lakes (Comte et al. 2018, Marois et al. 2022). Members of the phylum *Pseudomonadota* were detected in high abundance in all samples and include members of the family *Comamonadaceae* such as cold-tolerant *Polaromonas* species that are abundant in the Arctic hydrosphere and cryosphere, and well-adapted to cold conditions (Darcy et al. 2011, Gawor et al. 2016, Ciok et al. 2018, Girard et al. 2023). In the terrestrial habitats, communities were more diverse and less dominated by one or two phyla (both in DNA and RNA); therefore, although they were systematically detected, *Bacteroidota* and *Pseudmonadota* did not likely drive the whole community to the same extent as in marine and freshwater samples. There was a greater representation of members of the *Verrucomicrobiota, Cyanobacteria, Actinomycetota*, and *Myxococcota* phyla, which are all commonly found in Arctic lakes and soils (Cavaco et al. 2019, Colby et al. 2020, Marois et al. 2022), including in the water tracks over permafrost soils on WHI (Steven et al. 2013).

### Bacterial dispersal of WHL to terrestrial and marine environments

The DNA-based bacterial community composition diverged markedly across the sampled environments. Habitat type explained 28% of the total variation within bacterial community structure. As expected, each habitat was constrained by its specific ecological parameters, as shown by the differences in nutrients, carbon, and Chl-*a* levels. Terrestrial water samples were more heterogenous than the other types as shown in the nMDS. This result is consistent with the heterogeneity of sampled sites, since each water track differed in terms of bacterial mat abundance, water flow, depth, and length (Fig. S4).

Freshwater, terrestrial, and marine samples all had a comparable level of taxonomic richness. Shannon and Simpson diversity showed that bacterial communities among all habitats were diverse but had low evenness, and were dominated by one or few taxa. Oligotrophic conditions, cold temperatures, and the high UV irradiance regime of Arctic systems (Ji and Wei 2020) results in environmental stress and elevated competition within bacterial communities (Mallon et al. 2015, Holmberg and Jørgensen 2023, Malard et al. 2023) and dominance by fewer organisms.

Overall, only one phylotype was shared among the four habitats, indicating extremely limited bacterial dispersal among the habitats or a strong environmental filtering of microbes as they move among habitats across the watershed (Günther et al. 2016, Hauptmann et al. 2016, Žárský et al. 2018). This was unexpected since a previous study on the same lake and watershed revealed that >30% of the phylotypes were shared among the different sampled habitats (Comte et al. 2018). However, this study did not include measurements from the coastal embayment, and the timing and sampling of the water tracks was not the same. Water tracks are highly variable on a daily but also hourly cycle (Paquette et al. 2018), which means that the flow and bacterial load run-offs to the lake can also greatly vary based on sampling date and time. Another plausible cause for such divergence could be the short WRT in the moat section (∼10 days), which may quickly wash away incoming species from terrestrial habitats to the lake outlet. Therefore, terrestrial microorganisms might not have time to be incorporated within or disturb local bacterial communities in the main body of the lake before they are removed by physical and biological processes (Niño-García et al. 2016). While increasing the sampling effort and sequencing depth would likely improve the spatial resolution of habitat composition, and likely reveal interactions at a finer scale, our data shows that under current conditions, dispersal between habitats in WHL is limited. However, the low sequence yields observed in water tracks, particularly in terrestrial water, may have led to an overestimation of the proportion of unique ASVs detected in other habitats. While the estimated uncaptured ASVs for these samples using Chao1 indices revealed low prediction of missed richness (Table S7), increasing the filtered volume and the number of samples from this habitat may reveal a greater number of shared ASVs.

Among habitats, terrestrial water (water tracks) samples had the highest number of unique ASVs (33% of all ASVs) as seen in Comte et al. (2018). Terrestrial (snow and water tracks) and WHL samples shared 15 ASVs, which represented 0.9% of the total ASVs identified. Although low, this is the same proportion as observed by Comte et al. (2018) (25 operational taxonomic units representing 0.6% of the total). Marine and WHL samples shared seven ASVs, representing 0.5% of the total. Despite few shared phylotypes and no clear signature of the marine habitats on Ward Hunt bacterial communities, some key bacterial genera associated with marine habitats and aerosols were found in both WHL and terrestrial snow samples. Specifically, *Aquaspirillum arcticum* (see Butler et al. 1989), was abundant in the brackish proglacial bay, and was also detected (from rDNA) and active (from rRNA) in both WHL and terrestrial snow samples (Fig. S5). This may imply a cosmopolitan distribution of this taxon across the WHL, or may be indicative of some connectivity among the habitats. Among the *Oxalobacteraceae* family, the genus *Actimicrobium* was highly abundant in WHL samples but also in all marine and a few terrestrial samples. This genus has been isolated from Antarctic coastal sea water (Kim et al. 2011) and from the Arctic Ocean (Vipindas et al. 2023) highlighting the marine signature among the other samples. Low shared bacterial communities between marine and WHL could be attributable to the lake ice cover that acts as a physical barrier that limits the establishment of airborne microbes. However, ice-off events, such as those experienced on WHL in recent years (Bégin et al. 2021c, Paquette et al. 2015) and the annual presence of a large open moat may therefore be associated with an increased connectivity between the Arctic Ocean and coastal freshwaters. Seeding by marine species may also be exacerbated in the future by the ongoing loss of summer sea ice (Stroeve et al. 2025), and the resultant increase in open waters (Crawford et al. 2021) and ocean–atmosphere exchange. At a global scale, few bacterial taxa are found in both marine and freshwaters (McMahon and Newton 2024), and salinity will likely act as a strong environmental filter that minimizes the overlap in community composition between these biomes, irrespective of future connectivity.

More ASVs were shared between WHL and terrestrial samples than marine, highlighting a greater (but still low) degree of interaction between terrestrial habitats and WHL. Although there was strong hydrological connectivity between WHL and its watershed, as evidenced by the isotope and PARAFAC signatures, few bacterial taxa were shared between the lake and the catchment. This trend is in line with a previous study conducted in Greenland, which found little influence of glacier meltwater on a river bacterial community (Hauptmann et al. 2016). However, studies in the sub-Arctic in Nunavik and Hudson Bay showed that bacterial communities from two or more divergent habitats shared significantly more ASVs (Morency et al. 2022, Blais et al. 2024), indicating some site-specific variability.

The bacterial communities were phylogenetically distant between most habitat pairs. This divergence was less pronounced, however, for water from under the lake ice and terrestrial snow, which were phylogenetically closer than the other comparisons. This aligns with previous studies in the same watershed that identified snow as an important source of bacterial assemblages to the lake (Bégin et al. 2021c, Comte et al. 2018), and the likely flow of meltwaters directly beneath the ice due to convective circulation processes (Welch and Bergmann 1985, Jansen et al. 2021). Thus although terrestrial snow and WHL did not share a large core of bacterial phylotypes, the species that compose each community were phylogenetically closer than assemblages from other habitats.

### Autochthonous carbon supports bacterial communities among WHL system

The high correlation between marine bacterial communities and Chl-*a* suggests that autochthonous organic materials produced by the phytoplankton provide important substrates for bacterial growth in this habitat (Larsson and Hagström 1979, Cole 1982, Feltracco et al. 2021), either by exudation during growth or by organic release after cellular death (Cole 1982). The correlation between Chl-*a* and TP, DIC, and SUVA_254_ suggests that these variables also play a role in shaping the marine communities. In addition, C1 and C2 were also important variables, once combined to SUVA_254_ and DIC. This is consistent with a metagenomic study on marine microbes from >60 Arctic and Antarctic seawater samples that revealed microbes with diverse functions and able to produce and use many compounds (Cao et al. 2020).

The freshwater bacterial community of WHL was correlated with component C1, which is linked to tryptophan-like DOM. This relationship is constent with the central role of bacteria in producing and recycling nutrients and organic matter in Arctic food webs (Moran et al. 2000, Cammack et al. 2004). Among other tested variables, Chl-*a* and S_289_ also appeared to be drivers of WHL bacterial community. This combination of parameters is not surprising since all three of these variables (C1, Chl-*a*, and S_289_) are linked, as proxies for tryptophan-like DOM from algal production (Loiselle et al. 2009, Wauthy et al. 2018). This is consistent with a reliance by freshwater bacterial communities on internal production and recycling rather than allochthonous sources such as terrestrial organic matter.

The bacterial communities of terrestrial snow samples were correlated with component C2 and with SUVA_254_. These compounds are associated with the presence of aromatic terrestrial carbon and by freshly produced bacterial DOM. However, the weaker correlation (*ρ* = 0.25) suggests that other unmeasured, unknown factors also contribute to bacterial community assemblage. Snow is a heterogenous and dynamic matrix that accumulates particles and nutrients from atmospheric deposition (Brooks and Williams 1999, Kuhn 2001, Sickman et al. 2001). Consequently, there can be large differences among adjacent sites on the snowpack based on their bacterial or chemical content (Harding et al. 2011), and this large spatial variability likely contributed to the small effect of other tested variables in the statistical models for snow samples.

## Conclusions

Our analyses show that the bacterial communities in WHL and in nearby terrestrial and marine habitats are highly distinct despite their proximity and connectivity. Although there was a clear hydrological connection to the watershed, bacterial communities from WHL shared few phylotypes with the surrounding habitats, likely because of the strong environmental selection once the microbes enter new habitats. Based on the physicochemistry and the origin of carbon and nutrient sources, WHL is more strongly influenced by the terrestrial environment, which provides humic-like organic materials, as well as DIC and nutrients, to the lake. These inputs support and structure the phytoplankton and bacterial communities of WHL throught seasonal precipitation and runoff water. On the other hand, the coastal embayment and the offshore waters of the Arctic Ocean appeared to have only a limited bacterial influence on the lake mainly attributable to the still largely enduring ice conditions of the LIA, which limits the potential aerosolization of microbes from open sea water and their deposition onto ice-free lakes.

In summary, this study illustrates how bacterial assemblages can maintain similar levels of richness, yet greatly diverge in their community structure, even among adjacent, physically connected habitats. Our results show that despite the increasing openness and connectivity associated with climate warming, Arctic bacterial communities can be resilient to change and retain their large habitat-specific differences. However, much larger scale changes in temperature and precipitation are projected for the Arctic later this century (Stroeve et al. 2025), and it remains to be seen to what extent this resilience of Arctic microbial ecosystems can be maintained in the decades ahead.

## Supplementary Material

fiaf115_Supplemental_Files

## Data Availability

Water chemistry and limnological data are available in the environmental archive Nordicana D at https://nordicana.cen.ulaval.ca/en/publication.php?doi=45906CE-35C5B5D9CBF54114., and the 16S ribosomal RNA gene sequences are available on SRA in BioProject PRJNA1119994.
