# Supplementary material for: Bacterial divergence among the interconnected habitats of a High Arctic Lake
Source: FEMS Microbiol Ecol. 2025 Nov 18;101(12):fiaf115. doi: 10.1093/femsec/fiaf115 (PMC12663089; doi:10.1093/femsec/fiaf115)
Supplement: fiaf115_Supplemental_Files [file fiaf115_supplemental_files.zip › Blackburn-Desbiens_et_al._Supplementary_Data.docx]

***Bacterial divergence among the interconnected habitats of a High Arctic Lake***

**Authors**

Pénélope Blackburn-Desbiens^1,2,3^, Maxime Larose^1,2,3^, Raoul-Marie Couture^2,3,4^, Warwick F. Vincent^2,5,6^, Alexander I. Culley^2,7^, Catherine Girard^1,2,3,5^

**Supplementary Table S1.**

| **Material** | **Sample ID** | **Volume filtered**  **(ml)** | **Qubit concentration (ng/µL)** | **Input** | **Filtered** | **DenoisedF** | **DenoisedR** | **Merged** | **Nonchim** |
| --- | --- | --- | --- | --- | --- | --- | --- | --- | --- |
| DNA | Lagoon-ice-A | 1 060 | 0.55 | 55 649 | 24 276 | 23 597 | 23 690 | 17 703 | 9 513 |
|  | Lagoon-ice-B* | 1 282 | 1.88 | 587 | 239 | 209 | 186 | 117 | 117 |
|  | Lagoon-ice-C | 1 262 | 1.69 | 69 011 | 29 059 | 28 349 | 28 443 | 21 100 | 12 230 |
|  | Lagoon-water-A | 965 | 3.38 | 59 488 | 25 507 | 23 638 | 23 766 | 18 713 | 9 985 |
|  | Lagoon-water-B | 900 | 9.96 | 59 939 | 24 698 | 23 255 | 23 413 | 18 663 | 9 880 |
|  | Lagoon-water-C | 950 | 16.3 | 76 096 | 35 033 | 32 588 | 32 939 | 26 181 | 13 686 |
|  | Sea-ice-A | 1 590 | 2.9 | 59 110 | 28 994 | 28 817 | 28 826 | 26 702 | 23 157 |
|  | Sea-ice-B* | 755 | 0.316 | 564 | 237 | 213 | 216 | 157 | 153 |
|  | Sea-ice-C | 1 715 | 1.17 | 63 718 | 27 549 | 27 443 | 27 415 | 26 589 | 23 572 |
|  | Snow-WT-A | 420 | 0.518 | 57 682 | 20 938 | 16 502 | 16 532 | 11 345 | 10 123 |
|  | Snow-WT-B | 860 | 0.0364 | 50 470 | 17 527 | 12 716 | 12 756 | 5 150 | 4 297 |
|  | Snow-WT-C | 1 010 | 0.033 | 10 417 | 2 747 | 2545 | 2 218 | 1 950 | 1 888 |
|  | WH-T-R1 | 2 040 | 0.872 | 71 698 | 22 355 | 21 594 | 21 739 | 14 714 | 7 082 |
|  | WH-T-R2 | 4 150 | 0.68 | 61 827 | 22 589 | 21 809 | 22 099 | 15 339 | 6 946 |
|  | WH-T-R3 | 2 200 | 0.0658 | 76 978 | 27 709 | 26 962 | 27 213 | 19 862 | 10 006 |
|  | WH-M-R1 | 2 184 | 1.68 | 62 111 | 22 589 | 21 806 | 22 138 | 13 776 | 6 045 |
|  | WH-M-R2 | 3 315 | 0.0758 | 62 018 | 23 170 | 22 045 | 22 525 | 15 641 | 8 013 |
|  | WH-M-R3 | 1 780 | 0.0864 | 66 062 | 25 413 | 24 830 | 25 044 | 18 198 | 8 730 |
|  | WH-B-R1 | 915 | NA | 49 496 | 18 776 | 17 618 | 18 025 | 13 145 | 11 177 |
|  | WH-B-R2 | 982 | 0.0798 | 55 144 | 20 998 | 20 212 | 20 420 | 14 404 | 8 968 |
|  | WH-B-R3* | 1 304 | 0.013 | 0 | 0 | 0 | 0 | 0 | 0 |
|  | WH-0-R1 | 975 | 2.9 | 50 584 | 22 009 | 20 804 | 21 277 | 14 862 | 8 624 |
|  | WH-0-R2 | 1 021 | 9.94 | 63 457 | 25 235 | 23 895 | 24 236 | 15 041 | 9 136 |
|  | WH-0-R3 | 991 | 0.516 | 70 455 | 24 845 | 21 857 | 21 715 | 14 398 | 10 544 |
|  | WH-snow-A | 1 240 | 0.0576 | 59 339 | 21 444 | 19 607 | 19 517 | 13 578 | 8 150 |
|  | WH-snow-B | 1 210 | 0.0112 | 54 495 | 18 675 | 16 848 | 17 298 | 10,753 | 9 221 |
|  | WH-WT-site1-middle-A | 120 | 0.029 | 33 216 | 10 831 | 4 587 | 3 836 | 891 | 851 |
|  | WH-WT-site1-middle-B | 110 | 0.0304 | 43 329 | 15 235 | 6 977 | 6 713 | 1 583 | 1 558 |
|  | WH-WT-site1-Top-A | 120 | 0.014 | 45 773 | 16 539 | 8 930 | 8 625 | 2 247 | 2 232 |
|  | WH-WT-site1-Top-B | 116 | 0.039 | 33 892 | 12 063 | 5 762 | 4 938 | 932 | 829 |
|  | WH-WT-site2-middle-A | 94 | 0.24 | 29 250 | 8 768 | 3 674 | 3 468 | 774 | 739 |
|  | WH-WT-site2-middle-B | 104 | NA | 25 844 | 8 600 | 7 793 | 8 079 | 5 881 | 5 587 |
|  | WH-WT-Site2-Top-A | 93 | 0.0388 | 49 260 | 14 037 | 7 263 | 6 323 | 905 | 846 |
|  | WH-WT-Site2-Top-B | 103 | 0.0648 | 27 733 | 8 026 | 2 618 | 2 113 | 307 | 302 |
|  | WT-moat-A | 780 | 10.9 | 63 067 | 21 893 | 17 388 | 18 103 | 11 513 | 7 154 |
|  | WT-moat-B* | 800 | 21.6 | 2 313 | 72 | 11 | 18 | 0 | 0 |
|  | WT-moat-C* | 780 | 11 | 3 486 | 20 | 12 | 1 | 0 | 0 |

* Samples removed from the analysis due to low sequence counts.

**Supplementary Table S2.** Water residence time of Ward Hunt Lake.

| Ward Hunt Lake (pelagic) | WHL Moat |
| --- | --- |
| ${WRT}_{WHL}= \frac{\left( lake area \right)*average depth}{precipitation*\left( catchment \right)* evaporation}$ | ${WRT}_{Moat}= \frac{(moat area)}{50\% of WHL inflow}$ |
| ${WRT}_{WHL}= \frac{\left( 0.35*1 000 000 \right)*4}{0.158*\left( 1.82*1 000 000 \right)*0.5}$ | ${WRT}_{Moat}= \frac{\left( 800*5*0.5 \right)}{WHL inflow*0.5}$ |
| ${WRT}_{WHL}= 10 years$ | ${WRT}_{Moat}=10 days$ |

**Supplementary Table S3.**

| **Sample ID** | **Chl-*a***  **(µg L^-1^)** | **SUVA_254_** | **S_289_** | ***a*_320_**  **(m^-1^)** | **TP**  **(µg L^-1^)** | **TN**  **(µg L^-1^)** | **DIC**  **(mg C L^-1^)** | **DOC**  **(mg C L^-1^)** |
| --- | --- | --- | --- | --- | --- | --- | --- | --- |
| Lagoon-ice-A | 2.65 | 0.002 | 0.021 | 0.23 | <DL | 189 | 2.80 | 109 |
| Lagoon-ice-B | 1.82 | 0.002 | 0.027 | 0.24 | <DL | 127 | 1.61 | 147 |
| Lagoon-water-A | 2.18 | n/a | 0.018 | 0.51 | n/a | n/a | n/a | n/a |
| Lagoon-water-B | 1.09 | n/a | 0.020 | 0.54 | n/a | n/a | n/a | n/a |
| Sea-ice-A | 0.16 | 0.06 | 0.019 | 0.39 | <DL | 58.1 | 1.75 | 16.79 |
| Sea-ice-B | 0.15 | 0.006 | 0.035 | 0.11 | <DL | 60.6 | 1.74 | 29.4 |
| Sea-ice-C | 0.02 | n/a | n/a | n/a | n/a | n/a | n/a | n/a |
| Snow-WT-A | n/a | 24.22 | 0.008 | 2.98 | n/a | n/a | n/a | n/a |
| Snow-WT-B | n/a | 10.36 | 0.009 | 1.25 | n/a | n/a | n/a | n/a |
| Snow-WT-C | n/a | 8.44 | 0.008 | 0.99 | n/a | n/a | n/a | n/a |
| WH-T-R1 | 0.15 | 0.02 | 0.011 | 0.43 | <DL | 122 | 0.59 | 22.8 |
| WH-T-R2 | 0.10 | 0.008 | 0.016 | 0.31 | <DL | 38.7 | 0.83 | 28.4 |
| WH-M-R1 | 0.08 | 0.007 | 0.011 | 0.35 | <DL | 28.5 | 0.72 | 25.4 |
| WH-M-R2 | 0.08 | 0.01 | 0.009 | 0.49 | <DL | 20 | 0.76 | 122 |
| WH-B-R1 | 0.003 | 0.006 | 0.018 | 0.18 | <DL | 21.1 | 2.22 | 72.7 |
| WH-B-R2 | 0.07 | 0.02 | 0.010 | 0.51 | <DL | 38.6 | 2.27 | 57.1 |
| WH-0-R1 | 7.32 | 6.63 | 0.018 | 0.96 | 13.7 | 92.2 | 6.83 | 0.32 |
| WH-0-R2 | 6.58 | 1.88 | 0.023 | 0.56 | 17.7 | 127.2 | 3.94 | 0.17 |
| WH-0-R3 | 2.44 | n/a | n/a | n/a | n/a | n/a | n/a | n/a |
| WH-snow-A | 0.84 | 5.54 | 0.012 | 0.63 | n/a | n/a | n/a | n/a |
| WH-snow-B | 0.49 | 4.23 | 0.010 | 0.52 | n/a | n/a | n/a | n/a |

**Supplementary Table S4**

| **Sample ID** | **Bacteria**  **(cells/mL)** | **Viruses**  **(particles/mL)** |
| --- | --- | --- |
| Lagoon-ice-A | 62 789 | 110 500 |
| Lagoon-ice-B | 80 222 | 59 440 |
| Lagoon-water-A | 99 133 | 280 400 |
| Lagoon-water-B | 83 389 | 218 960 |
| Sea-ice-A | 16 267 | 89 500 |
| Sea-ice-B | 20 067 | 48 720 |
| Snow-WT-A | 161 378 | 114 400 |
| Snow-WT-B | 58 611 | 3 640 |
| WH-T-R1 | 8 311 | 74 800 |
| WH-T-R2 | 9 333 | 520 |
| WH-M-R1 | 7 167 | 69 700 |
| WH-M-R2 | 13 844 | 72 400 |
| WH-B-R1 | 8 267 | 73 200 |
| WH-B-R2 | 3 989 | 69 200 |

**Supplementary Table S5.**

| **Sample ID** | **δ^2^H** | **δ^18^O** |
| --- | --- | --- |
| Lagoon-ice | -69.41 | -8.87 |
| Lagoon-water | -164.59 | -21.22 |
| Sea-ice | -11.3 | -1.48 |
| Snow-WT | -186.67 | -24.00 |
| WH-B | -174.49 | -21.78 |
| WH-O | -172.43 | -21.89 |
| WH-T | -176.39 | -22.03 |
| WH-snow | -199.95 | -25.67 |

**Supplementary Table S6.**

| **Habitat community composition** | **Model** | **Spearman’s ρ** | **Tested variables** |
| --- | --- | --- | --- |
| Marine | 1* | 0.7717 | Chl-*a* |
|  | 2 | 0.6571 | Chl-*a*, C1 |
|  | 3 | 0.6571 | Chl-*a*, SUVA_254_, C2 |
|  | 4 | 0.6571 | Chl-*a*, SUVA_254_, C1, C2 |
|  | 5 | 0.6571 | Chl-*a*, SUVA_254_, C1, C2, DIC |
| Freshwater | 1* | 0.6601 | C1 |
|  | 2 | 0.5961 | Chl-*a*, S_289_ |
|  | 3 | 0.5982 | Chl-*a*, S_289_, C1 |
|  | 4 | 0.5906 | Chl-*a*, S_289_, C1, C2 |
|  | 5 | 0.5791 | Chl-*a*, S_289_, C1, C3, DIC |
| Terrestrial snow | 1 | 0.1636 | C2 |
|  | 2* | 0.2485 | SUVA_254_, C2 |
|  | 3 | 0.0788 | SUVA_254_, C2, C3 |
|  | 4 | 0.01881 | SUVA_254_, S_289_, C2, C3 |
|  | 5 | -0.1273 | SUVA254, S_289_, C1, C2, C3 |

* Best model

**Supplementary Table S7.**

| **Sample ID** | **Observed richness** | **Chao1 estimate** | **Missing richness (Chao1-Observed)** | **Percentage of missed ASVs (%)** |
| --- | --- | --- | --- | --- |
| Lagoon-ice-A | 70 | 78 | 8 | 11.4 |
| Lagoon-ice-C | 84 | 88 | 4 | 4.8 |
| Lagoon-water-A | 85 | 85.1 | 0.1 | 0.1 |
| Lagoon-water-B | 71 | 71.8 | 0.8 | 1.1 |
| Lagoon-water-C | 113 | 117 | 4 | 3.5 |
| Sea-ice-A | 32 | 32 | 0 | 0 |
| Sea-ice-C | 18 | 18.25 | 0.25 | 1.4 |
| Snow-WT-A | 25 | 26.125 | 1.125 | 4.5 |
| Snow-WT-B | 54 | 54.1 | 0.1 | 0.2 |
| Snow-WT-C | 25 | 25 | 0 | 0 |
| WH-R1-B | 72 | 72.9 | 0.9 | 1.3 |
| WH-R1-M | 38 | 38 | 0 | 0 |
| WH-R1-O | 41 | 41 | 0 | 0 |
| WH-R1-T | 33 | 33 | 0 | 0 |
| WH-R2-B | 47 | 47.6 | 0.6 | 1.3 |
| WH-R2-M | 49 | 49 | 0 | 0 |
| WH-R2-O | 44 | 44 | 0 | 0 |
| WH-R2-T | 34 | 43 | 9 | 26.5 |
| WH-R3-M | 37 | 37.4 | 0.4 | 1.1 |
| WH-R3-O | 74 | 74.13 | 0.13 | 0.2 |
| WH-R3-T | 36 | 36 | 0 | 0 |
| WH-snow-A | 44 | 44.4 | 0.4 | 0.9 |
| WH-snow-B | 97 | 97.75 | 0.75 | 0.8 |
| WH-WT-site1-middle-A | 42 | 42.28 | 0.28 | 0.7 |
| WH-WT-site1-middle-B | 42 | 42,35 | 0.35 | 0.8 |
| WH-WT-site1-Top-A | 82 | 83.26 | 1.26 | 1.5 |
| WH-WT-site1-Top-B | 40 | 40.5 | 0.5 | 1.3 |
| WH-WT-site2-middle-A | 25 | 25.11 | 0.11 | 0.4 |
| WH-WT-site2-middle-B | 68 | 68 | 0 | 0.0 |
| WH-WT-Site2-Top-A | 35 | 35.06 | 0.06 | 0.2 |
| WH-WT-Site2-Top-B | 13 | 13.1 | 0.1 | 0.8 |
| WT-moat-A | 42 | 42.9 | 0.9 | 2.1 |

**Supplementary Figure S1.**

**
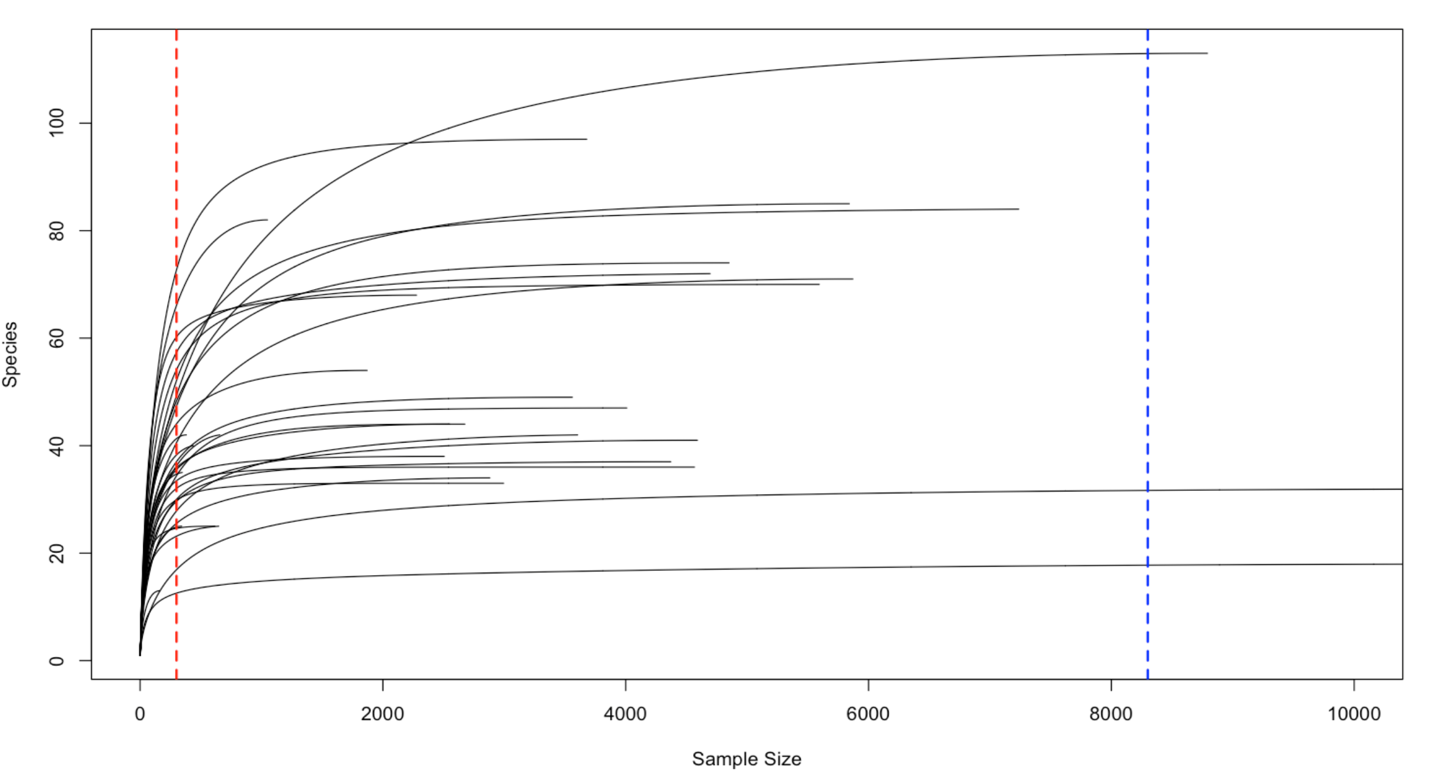
**

**Supplementary Figure S2.**

**Supplementary Figure S3.**

**Supplementary Figure S4.**

**Supplementary Figure S5.**
