# Supplementary material for: Bacterial divergence among the interconnected habitats of a High Arctic Lake
Source: FEMS Microbiol Ecol. 2025 Nov 18;101(12):fiaf115. doi: 10.1093/femsec/fiaf115 (PMC12663089; doi:10.1093/femsec/fiaf115)
Supplement: fiaf115_Supplemental_Files [file fiaf115_supplemental_files.zip › Blackburn-Desbiens_et_al._Supplementary_Data_caption.docx]

***Bacterial divergence among the interconnected habitats of a High Arctic Lake***

**Authors**

Pénélope Blackburn-Desbiens^1,2,3^, Maxime Larose^1,2,3^, Raoul-Marie Couture^2,3,4^, Warwick F. Vincent^2,5,6^, Alexander I. Culley^2,7^, Catherine Girard^1,2,3,5^

Supplementary Tables:

- **Supplementary Table S1**. Sequence processing, amplicon sequence variant (ASV) clustering and quality control. Input column represents the raw number of reads recovered from each sample before the cleaning and processing of sequences, Filtered represents the number of reads once the sequences are trimmed and filtered (primers removed), Denoised F and Denoised R show the number of quality-filtered reads, Merged shows counts of concatenated forward and reverse reads and Nonchim represents the number of reads following chimera removal.
- **Supplementary Table S2.** Water residence time of Ward Hunt Lake.
- **Supplementary Table S3.** Mean values for pelagic chlorophyll-*a* (Chl-*a*), specific ultraviolet absorbance at 254 nm (SUVA_254_), spectral slope at 289 nm (S_289_), absorption coefficient at 320nm (a320), total phosphorus (TP), total nitrogen (TN), dissolved inorganic carbon (DIC) and dissolved organic carbon (DOC) for each sample duplicate. Water tracks samples were not included because of a limited volume of water. Detection limits (DL) were of 8µg L^-1^ for TP, 20µg L^-1^ for TN and 0.05 mg C L^-1^ for DIC and DOC. Cells identified with n/a indicates no measurement.
- **Supplementary Table S4.** Flow cytometry counts for bacterial (cells/mL) and viral particles (particles/mL) for each duplicate of samples.
- **Supplementary Table S5.** Hydrogen and oxygen stable isotope ratios from each sample.
- **Supplementary Table S6.** Bioenv analysis of the microbial community composition of marine (n=4), freshwater (n=8) and terrestrial snow (n=4) habitats with overall best solution based on tested environmental variables.
- **Supplementary Table S7.** Estimation of the missing unsampled ASVs fraction from each sample based on the difference between Chao1 and Observed richness.

Supplementary Figures:

- **Supplementary Figure S1.** Rarefaction curves for Ward Hunt Lake samples based on bacterial 16S rRNA gene DNA. Red dotted line shows the 300 reads threshold of the lower used samples and blue dotted line shows the median.
- **Supplementary Figure S2.** Water chemistry profile in the water column of WHL in 2022. Profile was made through bore holes in lake ice at the deepest point of the water column.
- **Supplementary Figure S3.** Spectral characterization of dissolved organic matter (DOM) based on terrestrial snow, water under the ice, lake ice and marine samples. **A)** Absorbance at 320 nm **B)** SUVA at 254 nm **C)** Spectral slope at 289 nm. Different letters indicate statistically different concentrations between habitats (post-hoc Tukey, p <0.05).
- **Supplementary Figure S4.** Pictures of water tracks heterogeneity across the sampled terrestrial habitats, in the WHL watershed. Sampling points ranged from microbial mats and dark colored water (A and B) to less mats and almost clear water (C and D).
- **Supplementary Figure S5.** Relative abundance of bacterial genera from the *Olaxobacteraceae* family on DNA (**A**) and cDNA (**B**) (normalized and transformed data, Hellinger and log), for samples from marine (lagoon and sea ice), freshwater (lake water and ice), terrestrial (water tracks and snow) habitats on WHI. NA in the legend refers to non-assign bacterial genus, non-identified.
